# CD27 expression on Treg cells limits immune responses against tumors

**DOI:** 10.1007/s00109-021-02116-9

**Published:** 2021-08-23

**Authors:** Sabine Muth, Annekatrin Klaric, Markus Radsak, Hansjörg Schild, Hans Christian Probst

**Affiliations:** 1 Institute for Immunology, University Medical Center Mainz, Mainz, Germany; 2 Research Centre for Immunotherapy, University Medical Center Mainz, Mainz, Germany; 3 IIIrd Department of Medicine Hematology, Oncology, Pneumology, University Medical Center Mainz, Mainz, Germany

**Keywords:** Regulatory T cells, Immune evasion, Peripheral tolerance, Immunotherapy

## Abstract

**Abstract:**

Regulatory T cells (Tregs) suppress immune responses and thus contribute to immune homeostasis. On the downside, Tregs also limit immune responses against tumors promoting the progression of cancer. Among the many mechanisms implied in Treg-mediated suppression, the inhibition of dendritic cells (DCs) has been shown to be central in peripheral tolerance induction as well as in cancers. We have shown previously that the maintenance of peripheral T cell tolerance critically depends on cognate interactions between Tregs and DCs and that the CTL priming by unsuppressed steady state DCs is mediated via CD70. Here, we have investigated whether the CD70/CD27 axis is also involved in Treg-mediated suppression of anti-tumor immunity. Using a mixed bone marrow chimeric mouse model in which we can deplete regulatory T cells in a temporally controlled fashion, we show that Treg-expressed CD27 prevents the breakdown of peripheral tolerance and limits anti-tumor immunity. Furthermore, ablation of Treg expressed CD27 acts synergistically with PD-1 checkpoint inhibition to improve CTL mediated immunity against a solid tumor. Our data thus identify Treg-expressed CD27 as a potential target in cancer immunotherapy.

****Key messages**:**

- Treg expressed CD27 maintains steady state DC tolerogenic
- Treg expressed CD27 limits anti-tumor immunity
- Ablation of Treg expressed CD27 synergizes with PD-1 blockade to improve CTL mediated tumor control

**Supplementary information:**

The online version contains supplementary material available at 10.1007/s00109-021-02116-9.

## Introduction

Immune suppression by CD4^+^CD25^+^ FoxP3^+^ regulatory T cells (Treg) is indispensable for the maintenance of peripheral tolerance. Their central importance for immune homeostasis is evident in the fatal multi-organ autoimmune disease that is the consequence of impaired Treg development in humans and mice [1–3]. On the downside, Tregs also dampen immune responses against malignant cells and thus promote the progression of tumors [4]. High numbers of regulatory T cells and especially high Treg:CD8 + T cell ratios in the tumor microenvironment are associated with a poor prognosis in many malignancies [5], and it has been shown that Treg depletion results in reduced cancer progression or tumor rejection by the induction of CTL mediated immunity [6, 7]. The mechanisms used by regulatory T cells to inhibit immune responses are remarkably complex involving a wide variety of suppressive mechanisms and acting on many different cell types of both the innate and the adaptive immune system [8, 9]. It has become clear that inhibition of the function of antigen-presenting cells (APC) is a crucial mechanism of Treg-mediated suppression [9, 10]. Using a transgenic mouse model in which transgenic CTL epitope expression can be induced selectively on dendritic cells (DC) [11], we have shown that Treg depletion leads to a functional activation of dendritic cells resulting in priming of naive T cells instead of peripheral tolerance induction [12]. The suppression of dendritic cell activation by Treg cells thereby critically depends on direct TCR-MHC class II interactions between Tregs and DCs. DCs that lack MHC class II expression and thus cannot make cognate interactions with Treg cells show an activated phenotype and are completely unable to induce peripheral CD8^+^ T-cell tolerance resulting finally in the development of fatal, CTL mediated autoimmunity [13]. When we investigated the mechanisms that are involved in the breakdown of peripheral tolerance upon Treg depletion, we found that unsuppressed steady state DCs prime CTLs in a CD70-dependent manner. In line with previous published data demonstrating that overexpression of CD70 by dendritic cells alone breaks peripheral tolerance and induces autoimmunity, the injection of a blocking antibody to CD70 prevented CTL priming by unsuppressed DCs [14, 15]. Among the many suppressive mechanisms of Treg cells targeting DC function that have been described [16], it has been shown recently, that Treg downregulate CD70 from the plasma membrane of dendritic cells in a CD27-dependent manner [17]. However, the functional role of this pathway for the suppression of autoreactive CTL and immune responses against malignant cells remains undefined.  

Here, we have used a novel mouse model allowing the selective elimination of CD27-expressing Treg cells at a defined point in time to investigate the CD27-CD70 axis in Treg-mediated suppression of steady state DCs and anti-tumor immunity. We show that CD27 expression on regulatory T cells is critical for the induction of peripheral CD8 T cell tolerance in the immunological steady state but also limits anti-tumor immunity. Abrogation of CD27 expression on Tregs showed synergistically enhanced anti-tumor immunity unleashed by PD-1 blockade, suggesting that targeting Treg-expressed CD27 might be a promising therapeutic strategy to enhance the efficiency of checkpoint inhibition therapy.

## Material and methods

### Mice

DIETER double-transgenic mice allow tamoxifen-inducible presentation of three lymphocytic choriomeningitis virus (LCMV)-derived cytotoxic T lymphocyte (CTL) epitopes (GP_33–41_/ D^b^, GP_34–41_/K^b^, and NP_396–404_/D^b^) and one β-galactosidase-derived CTL epitope (β-gal_497–504_/^Kb^) by CD11c^high^ dendritic cells (DCs) [11]. FoxP3.LuciDTR-5 mice [6] allow the depletion of FoxP3 + T cells by the injection of diphtheria toxin. CD27 − / − mice [18] and RAG1^−/−^ mice [19] have been described previously. All mice were obtained from the central animal facility of Johannes Gutenberg University of Mainz and were bred and maintained under SPF conditions. All mice were on a C57BL/6 J background. Experiments were performed with age- and sex-matched mice and were conducted with permission of the Landesuntersuchungsamt Rheinland-Pfalz (Protocol G08-1–002).

### Generation of bone marrow chimeric mice

Bone marrow (BM) was isolated by flushing tibias and femurs of donor mice with PBS. BM cells were depleted of T cells using anti-mouse pan T Dynabeads (Invitrogen) according to the manufacturer’s instructions and mixed at the indicated ratios. A total of 4 × 10^6^ cells were injected i.v. into Rag1 − / − mice that had been lethally (6 Gy) irradiated using a ^137^Cs source. Mice were reconstituted i.v. with 70% BM from RAG1^−/−^  × DIETER mice and 15% BM from FoxP3.LuciDTR-5 (CD45.1 positive) mice and either with 15% BM from CD27 − / − mice or 15% BM from WT mice, together with 2 × 10^5^ CD25^+^ T cells from FoxP3.LuciDTR-5 mice, which had been isolated using a biotinylated antibody against CD25 (clone 7D4) and streptavidin (SA) microbeads and LS columns (Miltenyi), to prevent spontaneous autoimmunity [20]. For tumor experiments, Rag1 − / − mice were reconstituted i.v. with 50% BM from FoxP3.LuciDTR-5 (CD45.1 positive) mice and 50% BM from CD27 − / − together with 2 × 10^5^ CD25^+^ T cells from FoxP3.LuciDTR-5 mice. The following 3 weeks, mixed bone chimeric mice were given Borgal (1 mg/mL of sulfadoxin and 0.2 mg/mL of trimethoprim) in the drinking water and were rested for 8 weeks after transplantation before being used in experiments. Mixed BM cell populations were verified by flow cytometric analysis of CD45.1 and CD45.2 expression on blood T lymphocytes.

### Treatment of mice

Antigen presentation by steady-state DCs was induced in vivo by injecting DIETER BM chimeric mice i.p. with 2 mg of tamoxifen on day 0 [11]. WT FoxP3 + regulatory T cells were depleted by i.v. injection of 30 ng/g body weight of diphtheria toxin (Calbiochem) on days − 1, 0, 1, and 3. Depletion of all CD4^+^ T cells was achieved by i.v. injection of 0.5 mg of GK1.5 on day − 1*.* On day 8 after tamoxifen injection, the frequency of D^b^/GP_33–41_ specific CD8^+^ T cells in the blood was determined by staining with tetrameric MHC-peptide complexes*.*

Mixed bone marrow chimeric mice that have been injected with MC38 tumor cells were depleted of WT FoxP3 + regulatory T cells by i.v. injection of 30 ng/g body weight of diphtheria toxin (Calbiochem) on days 7 and 9 after tumor inoculation. PD-1 signaling pathway was blocked by i.v. injection of 250 µg anti-PD-1 (clone RMP1-14, BioXCell) on day 7 and day 9 after tumor inoculation. CD8^+^ T cells were depleted by the injection of 500 µg αCD8 (clone YTS169.4) i.v. on day 7 and day 9 upon MC38 inoculation.

### Cell lines

MC38 colon adenocarcinoma cells were thawed and cultured for 3 passages in DMEM (Thermo Fisher Scientific) supplemented with 1% penicillin–streptomycin, 10% FCS, 2 mM glutamine, and 1 mM sodium pyruvate before inoculation. MC38 tumor cells (1 × 10^6^ in 100 μl PBS) were injected subcutaneously into the right flank of WT + FoxP3-GDL5 or CD27 − / −  + FoxP3-GDL5 mixed chimeras. On day 7 and 9 after tumor inoculation, mice received 30 ng/g DT and/or 250 µg αPD-1 intravenously. In some experiments, mice additionally received 500 µg αCD8 i.v. Tumor growth was observed over a period of 15–21 days and survival was monitored until 35 days upon tumor inoculation. Tumor size was measured using a caliper. Mice were killed when there was external necrosis or when their tumor size reached more than 2 cm in any direction.

### Preparation of single-cell suspensions from MC38 tumors for flow cytometry

MC38 tumors were removed on day 15 after tumor inoculation and cut into small pieces. Tumors were digested using the dissociation of tumor tissue kit (Miltenyi) and a GentleMACS Dissociator (Miltenyi) according to the manufacturing protocol. Afterwards, tumor-infiltrating leukocytes (TILs) were enriched using CD45 TIL beads (Miltenyi) and LS Columns. Enriched TILs were stained with fluorochrome-coupled antibodies and flow cytometric analyzed.

### Antibodies and flow cytometric analysis

Antibodies against mouse CD4 (clone GK1.5) and CD8α (clone YTS196.4) were purified from hybridoma culture supernatant using protein G Sepharose (Genscript, Piscataway, NJ). Antibodies against PD-1 (clone RMP1.14) were purchased from BioXCell. Tetrameric peptide-MHC complexes were generated and staining was performed as described previously [21]. Fluorochrome-coupled antibodies for flow cytometry were purchased from eBioscience, BioLegend, or BD. Single-cell suspensions and enriched tumor-infiltrating leukocytes were incubated for 10 min with 0.5 mg/ml anti-CD16/CD32 (clone 2.4G2, produced in house) to block Fc-receptors followed by staining with fluorochrome-coupled antibodies for 20 min at 4 °C in FACS buffer (PBS 1% BSA, 20 mM EDTA). FoxP3 was detected using a FoxP3 Staining Kit (eBioscience) in accordance with the manufacturer’s instructions. Erythrocytes were lysed using red cell lysis buffer (RCLB). Samples were analyzed on either a LSR-II, FACSCanto II, or a FACSSymphony (Becton Dickinson, Mountain View, CA) and data were analyzed with FlowJo Analysis Software (Tree Star Inc, Ashland, OR).

### Intracellular cytokine staining

In order to detect IFNγ, TNFα, and Granzyme B produced by CD8^+^ T cells in tumor-infiltrating leukocytes (TILs), enriched TILs from MC38 tumors were incubated for 6 h at 37 °C with 0.5 mg/ml PMA (Sigma-Aldrich, St. Louis, MO) plus 0.05 mg/ml ionomycin (Sigma-Aldrich, St. Louis, MO) or with medium alone, in the presence of 5 mg/ml Brefeldin A (Sigma-Aldrich). Afterwards, cell surface markers were stained as described above and cells were fixed with 2% paraformaldehyde, permeabilized with 0.1% saponin (ROTH, Karlsruhe, Germany) in FACS buffer and stained intracellularly for the respective cytokines. Samples were analyzed on either a LSR-II, FACSCanto II, or a FACSSymphony (Becton Dickinson, Mountain View, CA), and data were analyzed with FlowJo Analysis Software (Tree Star Inc, Ashland, OR).

### Peptides

LCMV-derived peptide GP_33–41_ (KAVYNFATM, H-2Db) was kindly provided by Dr. Stefanovic, Interfaculty Institute for Cell Biology, Department of Immunology, University of Tübingen, Germany.

### Statistical Analysis

Statistical parameters including the exact value of n, the definition of center, dispersion, and precision measures (mean ± SD), and statistical significance are reported in the figures and figure legends. Data was judged to be statistically significant when *p* < 0.05 by two-tailed Student’s *t* test, one-way ANOVA with Bonferroni’s multiple comparisons correction, or log-rank (Mantle-Cox) test for survival analysis. In figures, asterisks denote statistical significance (*p < 0.05; **p < 0.01; ***p < 0.001; ****p < 0.0001). Statistical analysis was performed in GraphPad Prism.

## Results

### Mixed bone marrow chimeric mouse model for the conditional knock-out of specific functions in the Treg compartment

FoxP3^+^ regulatory T cells are essential guardians of immune homeostasis but also limit efficient immune responses to malignancies. A large number of molecular mechanisms by which Treg cells exert their suppressive function have been described whose relative contribution to immune suppression appears to be context dependent [22]. Conditional gene-deficient mice based on selective expression of Cre recombinase in Tregs have been developed, but in many cases, their use for studies in specific immune contexts such as immune responses to tumors have been hampered by systemic autoimmunity that results from inactivation of functionally important genes in Tregs.

**Fig. 1:**
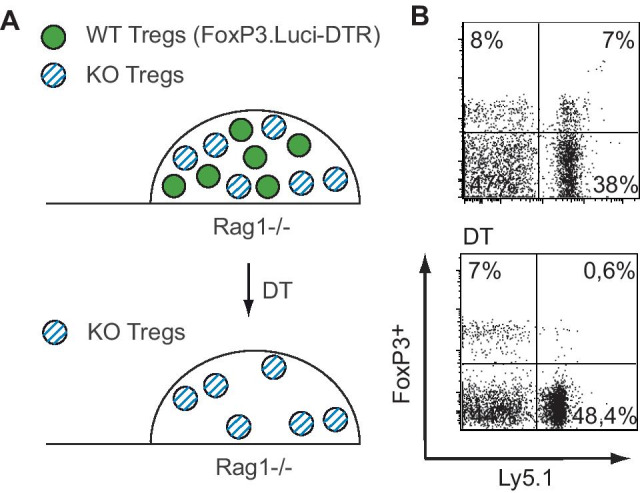
Model system of mixed bone marrow chimeric mice for the conditional knockout of specific functions in the Treg compartment. **A** Mixed bone marrow chimeric mice are generated by transplanting lethally irradiated Rag1 − / − mice with a mixture of 50% FoxP3.LuciDTR-5 (CD45.1, green circles) bone marrow and 50% bone marrow from mice deficient for a Treg effector molecule of interest (KO, CD45.2, blue striped circles). Administration of DT to these mixed chimeras results in the depletion of all DT-expressing Tregs of FoxP3.LuciDTR-5 origin. Remaining Treg will be all of the respective KO. **B** Mixed bone chimeric mice of 50% FoxP3.LuciDTR-5 (CD45.1) and 50% WT bone marrow (CD45.2) had been generated. Equal reconstitution of CD4^+^ FoxP3^+^ T cells was checked using CD45.1/2 congenic markers of the different Treg compartments (upper panel) in the blood. DT was injected into mixed chimeric mice on day 0 and day 2. Four days after DT treatment, distribution of CD45.1/2 expression on CD4^+^ FoxP3^+^ was analyzed in the blood by flow cytometry

**Fig. 2:**
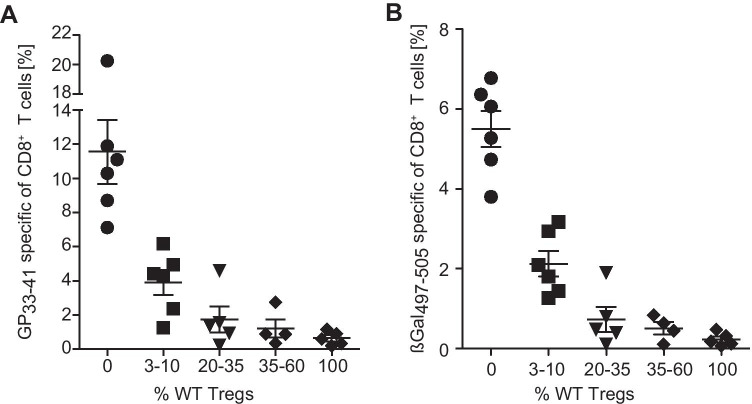
Depletion of up to 80% of Treg is possible without breakdown of peripheral CD8^+^ T cell tolerance. Mixed BM chimeric mice were generated by the reconstitution of lethally irradiated Rag1 − / − mice with BM cells from Rag1 − / − DIETER, FoxP3.LuciDTR-5 (CD45.1) and different amounts of BM cells from WT mice, resulting in chimeric mice with the indicated proportions of WT Tregs (0–100%). DTR-expressing Tregs were depleted on day − 1, 0, 1, and 3 by DT injection. On day 0, mice received tamoxifen i.p. to induce expression of CTL epitopes GP_33–41_ and ßGal_497–505_ on transgenic dendritic cells. At day 8, the frequency of CD8^+^ T cells specific for D^b^/GP_33–41_ (**A**) and K^b^/ßGal_497–505_ (**B**) in the blood was determined by staining with tetrameric MCH-peptide complexes. Data are representative of 2 independent experiments

To overcome this limitation and investigate Treg function in vivo without disturbing immune homeostasis, we have developed a mixed bone marrow chimeric mouse model where Treg lack a particular molecule in a temporally controlled fashion. We generated mixed bone marrow chimeras by reconstituting irradiated, T cell-deficient mice (to avoid residual radioresistant host Tregs) by transplanting a mixture of bone marrow from FoxP3.LuciDTR-5 mice ( Li et al. [6]) that express the diphtheria toxin receptor (DTR) under control of the FoxP3 promotor and bone marrow from mice deficient for the gene of interest. In these mixed bone marrow chimeric mice, half of all Treg express the DTR but are functionally wild type. As Treg suppression is a dominant mechanism, these wild-type Treg compensate for the deficiency in the Treg compartment of the specific knockout, allowing normal immune homeostasis. After the injection of DT into these mixed chimeric mice, DTR-expressing wild-type Treg are depleted and only knockout Treg remain, allowing to study the consequences of the knockout selectively in the Treg compartment (Fig. 1A). To check for equal reconstitution and to assess the efficiency of depletion, we made use of congenic CD45.1 expression on FoxP3.LuciDTR-5 deriving cells. Eight weeks upon reconstitution of 50% FoxP3.LuciDTR5 (CD45.1) and WT (CD45.2) mixed chimeras, we found a similar distribution of CD45.1 positive and negative CD4^+^ FoxP3^+^ cells indicating equal reconstitution of the Treg compartment. Upon DT injection, the population of DTR expressing Tregs was successfully depleted (Fig. 1B). To ensure that dominant tolerance was maintained after depletion of half of the Treg compartment, we used this conditional knockout model in combination the DIETER model of peripheral tolerance induction. Transgenic DIETER mice that allow tamoxifen inducible expression of transgenic CTL epitopes (GP_33–41_ and ßGal_497–505_) on dendritic cells [11], resulting in peripheral tolerance induction against the transgenic antigens if DCs are kept in a tolerogenic state by cognate interactions with functional Treg [13]. We generated mixed bone marrow chimeras by transplanting a mixture of 70% bone marrow from DIETER mice bred to RAG1-deficient mice plus different proportions of FoxP3.LuciDTR-5 bone marrow and bone marrow from WT mice (0–100% WT bone marrow as indicated in Fig. 2) into irradiated, RAG1-deficient hosts. The resulting mixed chimera express the DIETER transgene on DC upon tamoxifen injection but the T cells are derived with varying ratios from FoxP3.LuciDTR-5 (CD45.1) or WT (CD45.2) bone marrow cells. In line with our published work [13], we found that depletion of 100% Treg at the time of induction of the transgenic GP_33–41_ and ßGal_497–505_ CTL epitopes on dendritic cells resulted in a breakdown of peripheral tolerance, indicated by priming of CTL specific for the transgene-encoded epitopes. While some CTL priming was detectable in mice with up to 10% remaining WT Treg, no significant priming was detected when more than 20% of Treg remained undeleted, suggesting that as little as 20% Tregs are sufficient to control CTL priming by DCs (Fig. 2). Taken together, we have established a chimeric mouse model allowing us to study the role of various Treg effector molecules in vivo, in a temporally controlled fashion*,* without disturbing the homeostatic conditions of the immunological steady state.

### CD27 expression on regulatory T cells is essential for the maintenance of peripheral tolerance

**Fig. 3:**
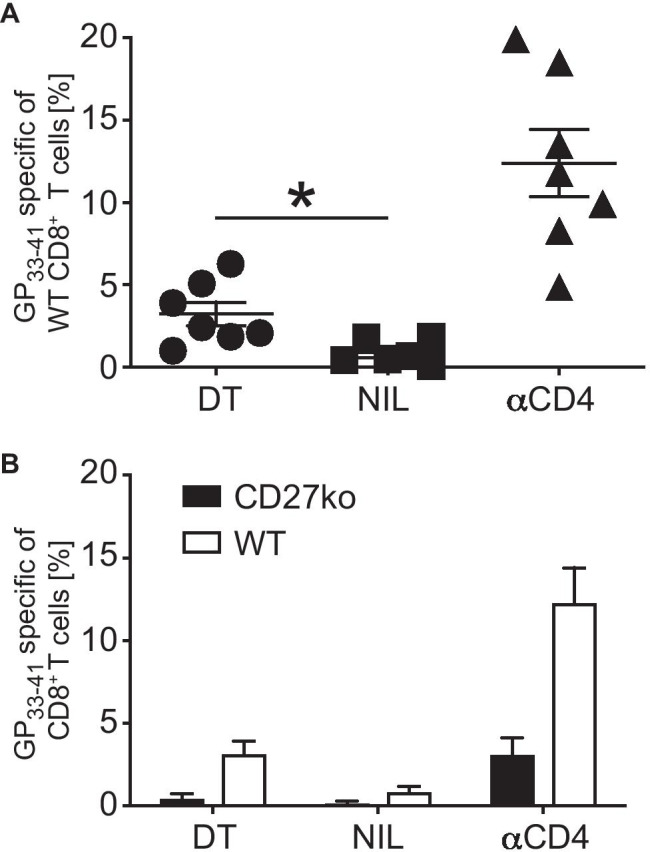
CD27 expression on regulatory T cells is necessary to maintain peripheral tolerance. Mixed BM chimeric mice were generated by reconstitution of lethally irradiated Rag1 − / − mice with a mixture of 70% BM cells from Rag1 − / − DIETER mice, 15% BM cells from FoxP3.LuciDTR-5 (CD45.1) mice, and 15% BM cells from CD27 deficient mice. **A** Reconstituted chimeric mice received either DT i.v. on day − 1, 0, 1, and 3 to deplete WT Tregs (of FoxP3.LuciDTR-5 origin) or were depleted of all Tregs by the injection of αCD4 i.v. on day − 1. As control, another group was left untreated. On day 0, all mice received 2 mg tamoxifen i.p. to induce antigen presentation on transgenic dendritic cells. At day 8, the frequency of CD8^+^ T cells specific for D^b^/GP_33–41_ in the blood was determined by staining with tetrameric MCH-peptide complexes. **B** Distribution of WT (open bars) and CD27 deficient (closed bars) primed D^b^/GP_33–41_ specific CD8^+^ T cells of the indicated groups described in **A**. Data are representative of 3 independent experiments. Horizontal bars represent mean ± SD (*n* = 7). Statistical significance was determined with Student’s *t* test *p $\le$ 0.05

We have previously shown, that Treg cells suppress CD8^+^ T cell priming by DCs by limiting co-stimulation through the CD70/CD27 axis [15]. Prompted by recent research demonstrating a role for CD27 in T cell suppression in vitro by mouse [17] and human [23] Treg, we set out to investigate whether CD27 expression on Treg cells is required for induction of peripheral tolerance by steady state DC. To this end, we used the conditional knockout model described above (Fig. 1) again in combination with the DIETER model. We created mixed bone chimeric mice by the reconstitution of lethally irradiated Rag-deficient mice with a mixture of bone marrow cells isolated from Rag − / − DIETER, FoxP3.Luci-DTR (CD45.1) and CD27-deficient mice. The resulting chimeric mice express the DIETER transgenic CTL epitopes on dendritic cells upon tamoxifen injection whereas the T cells are derived in equal ratios from FoxP3.Luci-DTR and CD27 − / − bone marrow cells. Flow cytometric analysis of splenic Treg cells revealed no obvious phenotypical difference between CD27 − / − and WT Treg cells (Supplemental Fig. 1). After reconstitution, mixed chimeras were injected with tamoxifen (TAM) to induce presentation of CTL epitopes on transgenic dendritic cells. Some mice received multiple injections with DT that resulted in the depletion of FoxP3.Luci-DTR Tregs or mice were depleted of all Tregs by the injection of αCD4 antibody. The frequency of LCMV GP_33–41_ specific T cells was measured 8 days after tamoxifen injection. As above, antigen presentation by steady state DCs did not induce expansion of antigen specific CD8^+^ T cells when 50% of WT Treg were present (Fig. 3A, NIL) and massive expansion of specific CD8^+^ T cells when all Treg were depleted (Fig. 3A, αCD4). Chimeric mice in which WT Tregs had been depleted by DT application, leaving only Tregs deficient for CD27, showed a significant expansion of antigen specific CD8^+^ T cells (Fig. 3A, DT) suggesting that CD27 expression on Tregs is required to keep steady state DC in a tolerogenic state. In line with previously published work on the importance of CD27 expression on CD8^+^ T cells for CTL priming [24], we found the primed CD8^+^ T cells to be almost exclusively derived from the CD27 competent (CD45.1) bone marrow. (Fig. 3B),

### CD27 expression on regulatory T cells promotes tumor growth

**Fig. 4:**
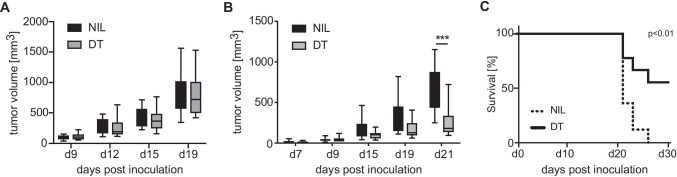
CD27 expression on regulatory T cells promotes tumor progression. Irradiated RAG1 − / − mice were reconstituted with a mixture of bone marrow cells containing 50% FoxP3.LuciDTR-5 (CD45.1) cells and 50% cells from either **A** WT mice or **B** mice deficient for CD27. Eight weeks after reconstitution, mice were injected with 10^6^ MC38 tumor cells in the right flank. Seven and nine days after tumor inoculation, mice were treated with 30 ng/g DT i.v. (gray bars) or left untreated (closed bars) and tumor growth was measured over time. **C** Cumulative survival curve of mice in **B**. Data are representative of 3 independent experiments. Boxes and whiskers depict interquartile range and range of data (**A**, *n* = 14; **B** and **C**, *n* = 10). Statistical significance was determined with Student’s *t* test or log-rank (Mantle-Cox) test for survival analysis ***p $\le$ 0.001

To address a potential role of CD27 expression on Tregs in inhibiting anti-tumor immunity, we used a transplantable adenocarcinoma model to challenge mice with a conditional deficiency of CD27 in the Treg compartment. As described before, we generated mixed bone chimeric mice by the reconstitution of lethally irradiated Rag1 − / − deficient mice with equal proportions of FoxP3.Luci-DTR and CD27 − / − bone marrow cells. After complete reconstitution, mice were transplanted subcutaneously with MC38 colon adenocarcinoma cells. When the tumor was palpable, two doses of DT were injected to deplete FoxP3.Luci-DTR + Tregs and tumor growth was measured over time and survival monitored. To exclude that depletion of half of the Treg compartment results in the rejection of tumor cells, we additionally generated mixed bone chimeric mice of FoxP3.Luci-DTR and WT bone marrow cells. As expected, we observed a rapid outgrowth of MC38 tumors in untreated mixed chimeras (Fig. 3A, B, black). Tumor volume of DT treated FoxP3.Luci-DTR + WT mixed chimeras was comparable to untreated chimeras indicating that depletion of 50% of Tregs had no effect on tumor outgrowth (Fig. 4A). In untreated FoxP3.Luci-DTR + CD27 − / − , mixed bone chimeric MC38 tumor cells also grew out and mice died within 27 days upon tumor inoculation (Fig. 4B, C). However, depletion of FoxP3.Luci-DTR Tregs from FoxP3.Luci-DTR + CD27 − / − chimeras by DT injection resulted in a significant reduction in tumor growth and prolonged survival (Fig. 4B, C), indicating that CD27 expression on regulatory T cell inhibits immune responses against MC38 tumor cells.

### PD-1 blockade and CD27 deficiency on Treg cells synergize to limit tumor growth

**Fig. 5:**
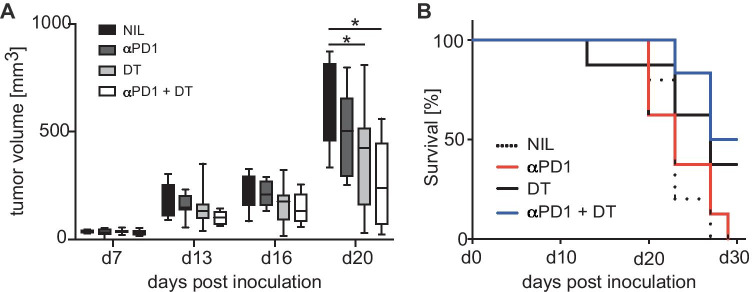
PD-1 blockade and CD27 deficiency on regulatory T cells synergizes to limit tumor growth. Irradiated RAG1 − / − mice were reconstituted with an equal mixture of bone marrow cells containing 50% FoxP3.LuciDTR-5 (CD45.1) cells and 50% cells from mice deficient for CD27. Eight weeks after reconstitution, mice were injected s.c. with MC38 tumor cells. Seven and nine days after tumor inoculation, mice were treated either with 30 ng/g DT i.v., with 250 µg a-PD-1 i.v. or with a combination of DT and a-PD-1. As for the control, some mice were left untreated. **A** Tumor growth was measured over time. **B** Cumulative survival curve of mice described in **A**. Data are representative of 3 independent experiments. Boxes and whiskers depict interquartile range and range of data (**A**, *n* = 14; **B** and **C**, *n* = 10). Statistical significance was determined with one-way ANOVA with Bonferroni’s multiple comparisons correction *p < 0.05

PD-1 is expressed on activated T cells and limits lymphocyte proliferation and function to prevent overwhelming immune reactions and to maintain peripheral tolerance [25, 26], but PD-1 also limits T cell responses against tumors. Nowadays, antibodies against PD-1 signaling pathways are part of the standard therapy for several malignancies although not all patients benefit from anti PD-1 or anti PDL-1 treatment [27]. Given the impact of Treg-derived CD27 on tumor growth (Fig. 4B, C), we questioned whether PD-1 blockade and abrogation of CD27 in the Treg compartment at the same time might have synergistic effects on antitumor immunity. To address this, we transplanted FoxP3.Luci-DTR + CD27 − / − mixed bone marrow chimeric mice subcutaneously with MC38 adenocarcinoma cells. When palpable tumors had developed, mice were either left untreated, or injected with DT, to deplete FoxP3.Luci-DTR + Tregs or with anti-PD-1 antibody to block the PD-1 signaling pathway or with both, anti-PD-1 and DT. DT injection alone resulted in a reduced tumor outgrowth and in an improved survival compared to untreated controls as observed before (Figs. 4B, C and 5). As expected, PD-1 blockade also resulted in a delayed tumor growth although this did not reach statistical significance at the group size studied. Combined injection of anti-PD-1 antibodies and DT improved the efficacy of both single treatments. Tumor growth was clearly reduced and survival of the mice was prolonged compared to untreated controls (Fig. 5) suggesting a synergistic effect of CD27 deficiency in the Treg compartment and blockade of the PD-1 signaling pathway on antitumor immunity.

**Fig. 6:**
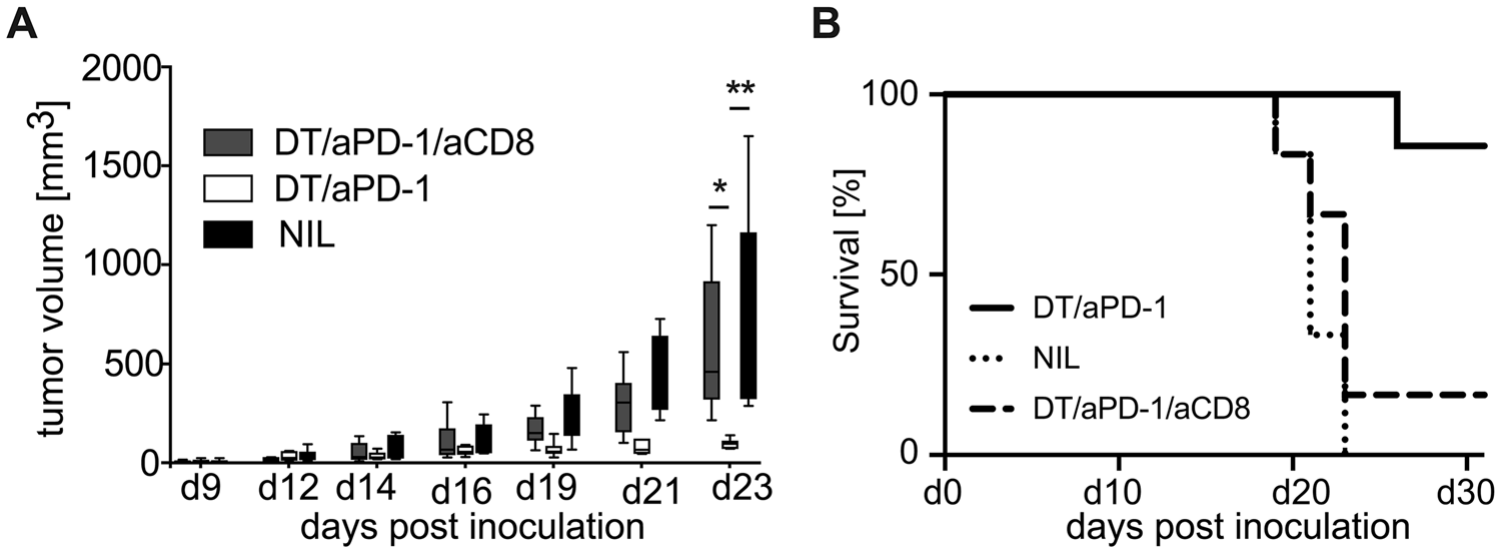
CD8^+^ T cells mediate Tumor control after PD-1 blockade and induction of CD27 deficiency on regulatory T cells. Irradiated RAG1 − / − mice were reconstituted with an equal mixture of bone marrow cells containing 50% FoxP3.LuciDTR-5 (CD45.1) cells and 50% cells from deficient mice. Eight weeks after reconstitution, mice were injected s.c. with MC38 tumor cells. Seven and nine days after tumor inoculation, mice were treated with DT i.v. together with αPD-1 i.v. or with DT and αPD-1 in combination with αCD8. As for the control, some mice were left untreated. **A** Tumor growth was measured over time. **B** Cumulative survival curve of mice described in **A**. Data are representative of 2 independent experiments. Boxes and whiskers depict interquartile range and range of data (*n* = 6). Statistical significance was determined with one-way ANOVA with Tukey’s multiple comparisons correction or log-rank (Mantle-Cox) test for survival analysis **p < 0.005; *p < 0.05

Consistent with the impaired induction of peripheral CD8^+^ T cell tolerance we had observed in the DIETER model, we found synergistic tumor control upon abrogation of CD27 expression in the Treg compartment and PD-1 blockade to be entirely dependent on CD8^+^ T cells (Fig. 6).

### Treg-derived CD27 and PD-1 signaling limits tumor infiltration of CD8^+^ T cells and CTL effector functions

**Fig. 7:**
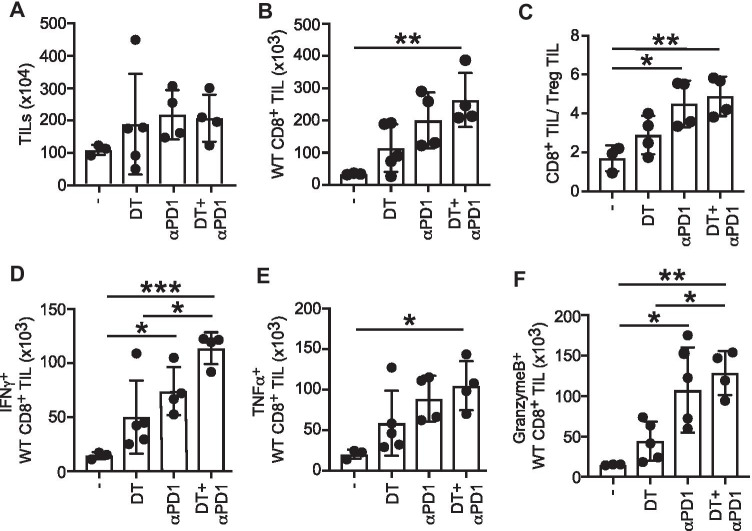
Treg derived CD27 and PD-1 signaling limits tumor infiltration of CD8^+^ T cells and CTL effector functions. Mixed BM chimeric mice were generated by the reconstitution of lethally irradiated mice with equal numbers of FoxP3.LuciDTR-5 (CD45.1) cells and CD27 − / − BM cells. Eight weeks upon reconstitution, mice were injected s.c. with MC38 tumor cells. Seven and nine days after tumor inoculation, mice were treated either with DT i.v. or with αPD-1 i.v. or with a combination of both. As for the control, some mice were left untreated. Tumors were removed 15 days after inoculation. Tumors were digested and tumor-infiltrating lymphocytes (TILs) were enriched using CD45 beads. Total numbers of all TILs (**A**), of WT CD8^+^ TIL (**B**), and CD8^+^ T cells/ Treg TIL ratios (**C**) were determined by flow cytometric analysis. TILs were restimulated with PMA + Ionomycin for 4.5 h and IFNγ (**D**), TNFα (**E**), and Granzyme B (**F**) production of wt CD8 + TILs were determined using flow cytometry. Data of 2 independent experiments are shown. Horizontal bars represent mean ± SD (*n* = 4; DT, *n* = 5; untreated, *n* = 3). Statistical significance was determined with one-way ANOVA with Bonferroni’s multiple comparisons correction *p < 0.05, **p $\le$ 0.01, ***p $\le$ 0.001

To get mechanistic insights into the role of Treg-derived CD27 with combinatorial PD-1 signaling blockade on CD8^+^ T cell mediated antitumor immunity, we analyzed numbers and effector functions of tumor infiltrating CD8^+^ T cells in MC38-bearing FoxP3.Luci-DTR5 + CD27 − / − mixed chimeric mice. MC38 tumor cells were inoculated sc. in reconstituted mixed chimeras and the WT Treg compartment was depleted by the injection of diphtheria toxin. Additionally, we injected DT in combination with anti-PD-1 antibody to assess synergistic effects of Treg-derived CD27 with concomitant blockade of the PD-1 signaling pathway on CD8^+^ T cell numbers and effector functions within the tumor. There was no difference in the total number of isolated tumor-infiltrating leukocytes (TILs) among the different treated groups (Fig. 7A), and also the overall numbers of B cells, NK cells, and monocytic TIL populations remained unchanged (Supplemental Fig. 2). A tendency for increased CD8^+^ T cell numbers was observed in both single-treated groups compared to untreated controls, although this did not reach statistical significance. However, combined PD-1 blockade and DT injection resulted in a substantial increase in tumor infiltrating CD8^+^ T cells underlining the synergistic effect of combinatorial PD-1 signaling blockade and CD27 deficiency on Tregs (Fig. 7B). Besides the accumulation of CD8^+^ TILs, there was also a significant increase in the CD8^+^:Treg ratio within the tumor microenvironment upon combined DT and aPD-1 treatment (Fig. 7C). As our data demonstrates that abrogation of Treg CD27 in combination with PD-1 signaling blockade affects CD8^+^ T cells numbers within the tumor, we next assessed effector function of intratumoral CD8^+^ T cells. Therefore, we stimulated TILs with PMA-Ionomycin and intracellularly stained CD8^+^ T cells for IFNγ, TNFα, and Granzyme B expression (Fig. 7D, E, F). Tumor-infiltrating CD8^+^ T cells from aPD-1 single-treated mice showed an increase of IFNγ (Fig. 7D), TNFα (Fig. 7E), and Granzyme B expression (Fig. 7F) compared to untreated controls. Although, to a lesser extent, this was also observed in DT treated mice (Fig. 7D, E, F). However, combined DT and αPD-1 treatment synergizes to improve CTL effector functions compared to both single treatments (Fig. 7D, E, F). Our findings thus demonstrate an important role for Treg derived CD27 in suppressing CD8^+^ T cell mediated antitumor immune responses by limiting intratumoral CD8^+^ T cell numbers and effector functions.

Collectively, our data demonstrate the involvement of CD27 expression by limiting immune responses against tumors and suggest abrogation of Treg-derived CD27 as a suitable strategy to improve the efficacy of PD-1 checkpoint inhibition therapy.

## Discussion

CD4^+^ FoxP3^+^ regulatory T (Treg) cells promote tumor growth by inhibiting anti-tumor immune responses and cancer immune surveillance [28, 29]. Consequentially, the molecular mechanisms of immune suppression by Tregs represent promising targets in tumor therapy. Over the years, a considerable number of such suppressive mechanisms have been described, in most cases, due to their involvement in suppression of immune responses by Tregs in vitro. [30, 31]. The development of mice expressing Cre recombinase under the control of the FoxP3 promoter [32, 33] has made selective gene targeting in Tregs possible and thus created the opportunity to study the molecular mechanisms of Treg suppression in vivo*.* However, using this strategy, targeted genes become deleted already during Treg development at the moment transcription of FoxP3 is initiated. As a consequence, FoxP3 targeted deletion of any gene that is essential for Treg function, or even for cell function and survival in general, will result in impaired Treg function and generalized autoimmunity already in early life [32, 34–37], hindering studies of Treg function in specific contexts, such as immune escape of tumor. We describe here a model that allows the temporally controlled ablation of immune functions in the Treg compartment and thus overcomes this limitation. By generating mixed bone marrow chimeras which carry a gene deficiency in only half of the Treg compartment whereas the other half of Tregs are functionally wild type, we ensure normal immune homeostasis and, in line with previous work [6], we demonstrate that as few as 20% of wild-type Tregs are sufficient to maintain peripheral tolerance. Depletion of Tregs in vivo has been shown to generate an enormous pressure for homeostatic expansion on remaining Tregs, which rapidly replenish the Treg compartment [38]. In the mixed bone marrow chimeric situation used here, we observed that the pressure generated by depletion of the WT half of Tregs is largely absorbed by proliferation of the remaining Tregs, which are deficient for a gene of interest, allowing to create a conditional knock out in the Treg compartment for at least 2 weeks (data not shown). On the downside, before depletion, KO Tregs in this model have been developed in competition with WT Tregs. As a result, gene deficiencies that confer a strong competitive disadvantage, such as deficiencies in genes necessary for differentiation and survival of Tregs, cannot be studied in this mixed chimeric model.

In the present study, we have used our conditional knockout model to explored the contribution of Treg expressed CD27 on suppression of immune responses to tumors. CD27, a member of the TNF receptor superfamily, is a key T cell costimulatory receptor [39]. Ligation of CD27 by CD70 on APC promotes T cell proliferation, differentiation, and survival [40]. In particular, CD27 ligation has been shown to promote T_h_1 [41] and CTL [42] priming. However, CD27 is expressed to higher levels on Tregs than conventional T cells in naïve mice [17] and CD27 expression on human CD4^+^CD25^+^ T cells from peripheral blood and from the synovia of idiopathic juvenile arthritis patients has been shown to correlate with suppressive activity [43, 44], suggesting a role of CD27 for Treg function. Indeed, CD27 on Tregs cells was found to downregulate the priming of Th1 responses by DCs [17]. In line with these results, we show here that ablation of CD27 selectively in the Treg compartment impaired peripheral tolerance induction by steady state DC and allowed for CD8^+^ T cell priming in the steady state. We have previously demonstrated, that CTL priming by DC that results from impaired Treg suppression depends on costimulation through the CD70/CD27 axis and interpreted enhanced CD70 co-stimulation as a consequence of a heightened activation state of steady state DC in the absence of suppression by Tregs [15]. However, work by Dhainaut et al. showing that ligation of CD70 on DC by CD27 expressed on Treg cells results in removal of CD70 on the DCs plasma membrane through internalization [17], suggests a more direct mechanism by which Tregs can suppress co-stimulation through the CD70/CD27 axis.

Using the mixed bone chimeric model described here, that allows the ablation of CD27 expressing T cells at a defined point in time allowed us to study the involvement of Treg-expressed CD27 in suppression of immunity to an already established and growing tumor. Ablation of CD27 in the Treg compartment suppressed the growth of established tumors and prolonged survival. Ochsenbein and colleagues had previously demonstrated a reduced tumor growth and an increased apoptosis of tumor infiltrating Tregs in CD27 − / − mice [45]. Our results here underpin their notion that CD27 expression on Tregs supports tumor growth.

The tumor model used here, MC38 colon adenocarcinoma, has been shown to have a high mutational load [46] and to express PDL-1 on tumor cells and the tumor microenvironment [47] and has therefore extensively been used as a target model for immunotherapy involving PD-1 or PD-L1 blockade. We show here, that the suppression of tumor growth resulting from ablation of CD27 in the Treg compartment, is strongly synergistically supported by blockade of PD-1. Mice that received both CD27 Treg ablation and PD-1 blockade showed enhanced infiltration of CD8^+^ T cells into the tumor and those cells displayed enhanced functionality. Tumor control by CD27 Treg ablation and PD-1 blockade was completely abolished by depletion of CD8^+^ T cells. Our finding here showing that CD27 expression on Tregs is involved in peripheral CD8^+^ T cell tolerance induction together with the important role of PD-1 on CD8^+^ T cells in this process previously described by us [26], implicate CTLs as a target for synergistic ablation of Treg CD27 and PD-1 blockade.

Previous studies have shown that direct CD27 ligation synergizes with PD-1/PDL1 blockade to unleash CD8^+^ T cell driven anti-tumor immunity [48, 49]. Our work here suggests that blocking of CD27 selectively on Tregs might be a therapeutic strategy for immunotherapy of cancer in synergy with blockade of the PD-1 PD-L1 checkpoint.

## Supplementary information

Below is the link to the electronic supplementary material.

Supplementary file1 (PDF 179 KB)

## Data Availability

All data generated or analyzed during this study are included in this published article [and its supplementary information files].
