# Supplementary material for: CD27 expression on Treg cells limits immune responses against tumors
Source: J Mol Med (Berl). 2021 Aug 23;100(3):439–49. doi: 10.1007/s00109-021-02116-9 (PMC8843905; doi:10.1007/s00109-021-02116-9)
Supplement: Supplementary file 1 — Supplementary file1 (PDF 179 KB) [file 109_2021_2116_MOESM1_ESM.pdf]

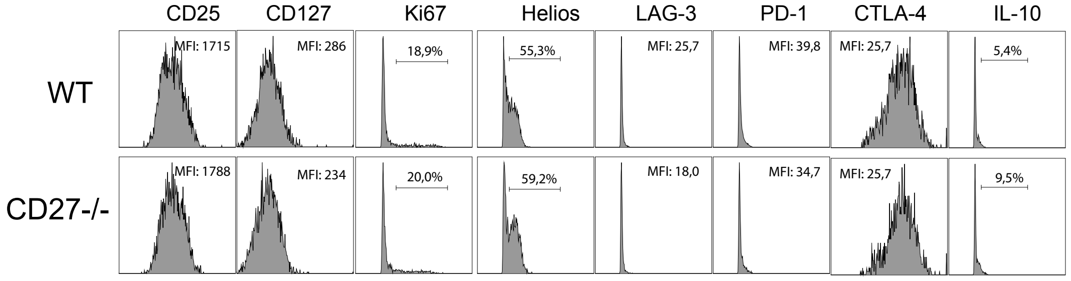

Supp. Figure 1: Phenotype of splenic CD27-/- Treg
Splenocytes of CD27-/- and WT mice were stained on the surface for CD4, CD25, CD127, CTLA-4, PD-1, LAG-3 and intracellularly for FoxP3, Ki67 and Helios. Intracellular staining for IL-10 was done after 5h stimulation with PMA and Ionomycin in the presence of BrefeldinA. All plots are gated on CD4+ FoxP3+ cells. MFI: median fluorecence intensity. Data of one representative of four mice per group are shown.

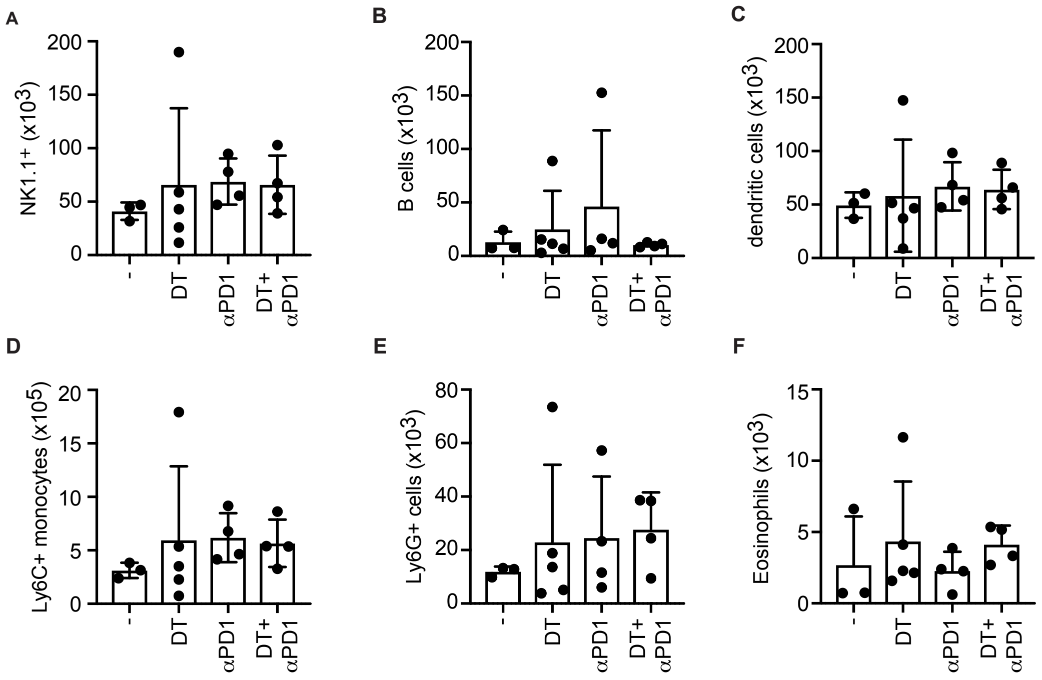

Supp. Figure 2: Tumor infiltrating leukocytes in MC38 bearing FoxP3.Luci-DTR5 x CD27-/- mixed chimeras

Mixed BM chimeric mice were generated by the reconstitution of lethally irradiated mice with equal numbers of FoxP3.LuciDTR-5 (CD45.1) cells and CD27-/- BM cells. 8 weeks upon reconstitution, mice were injected with 106 MC38 tumor cells into the right flank. 7 and 9 days after tumor inoculation, mice were treated either with 30ng/g DT iv or with 250 µg -PD1 i.v. or with a combination of both. As control some mice were left untreated. Tumors were removed 15 days after inoculation. Tumors were digested and tumor infiltrating leukocytes (TILs) were enriched using CD45 beads. Total numbers of NK1.1 cells (A), B cells (B) dendritic cells (C), Ly6G+ monocytes (D), Ly6C monocytes (E) and eosinophils (F) were determined using flow cytometry. Data of 2 independent experiments are shown. Horizontal bars represent mean ± SD (n= 4; DT, n= 5; untreated, n=3).
